# Analysis of ICU physician predictions for ICU length of stay

**DOI:** 10.1186/cc14671

**Published:** 2015-09-28

**Authors:** Antonio Paulo N Junior, Pedro Caruso

**Affiliations:** 1 A.C. Camargo Cancer Center, Liberdade, São Paulo, São Paulo, Brazil

## Introduction

An adequate prediction of ICU length of stay (ICU LOS) is of paramount importance to adequately allocate resources and inform patients and families. However, literature evaluating ICU physician predictions on ICU LOS are sparse.

## Methods

Patients admitted to four medical-surgical ICUs in an oncology teaching hospital in 2014 were included. As part of the admission form, the physician responsible for patient admission is asked to inform an estimate of ICU LOS for that patient (less than 48 hours, 2-5 days or more than 5 days). Agreement of predicted and actual ICU LOS was calculated. Patients and physician characteristics at admission were evaluated to identify associated factors for underestimation and overestimation of ICU LOS. Two logistic regression analyses were performed to identify independent risk factors for each outcome.

## Results

A total of 2955 patients were admitted during the study period (female sex: 46.5%, median age: 61 (51-71) years). Admissions were mainly for elective surgery (56.5%), followed by medical reasons (37%). Readmissions encompassed 9.3% of total admissions. Median SAPS 3 was 48 (40-64) and ICU mortality was 8.5%. Median ICU LOS was 2 (1-3) days. Physicians adequately predicted ICU LOS in 53% of admissions. ICU LOS were underestimated in 29% and overestimated in 18% of cases. Kappa statistics was 0.222 (0.195-0.249). Sex, scheduled surgical admission, Eastern Cooperative Oncology Group (ECOG) performance status, mechanical ventilation, vasopressor use and active infection at admission were associated with underestimation of ICU LOS. Male sex (OR = 0.80; 95% CI 0.65-0.98), and active infection at admission (OR = 1.35; 95% CI 1.02-1.78) were independently associated with underestimation of ICU LOS in logistic regression. Type of admission (medical and urgent surgery), reason for admission (not postoperative monitoring), ECOG, mechanical ventilation, vasopressor use, delirium and infectious status at admission, SAPS 3, serum creatinine and being readmitted were associated with overestimation of ICU LOS. Type of admission (OR = 0.64; 95% CI 0.51-0.80), reason for admission (OR = 0.93; 95% CI 0.87-0.99), ECOG (OR = 0.84; 95% CI 0.76-0.92) and active infection at admission (OR = 0.60) were independently associated with overestimation of ICU LOS in logistic regression.

## Conclusion

ICU physicians adequately predicted ICU LOS in only 53% of admissions. Sex and active infection were independently associated with underestimation of ICU LOS. Type of and reason for admission, ECOG, ventilator support and active infection at admission were independently associated with overestimation of ICU LOS.

